# Role of Type 4B Secretion System Protein, IcmE, in the Pathogenesis of *Coxiella burnetii*

**DOI:** 10.3390/pathogens13050405

**Published:** 2024-05-14

**Authors:** Rajesh Palanisamy, Yan Zhang, Guoquan Zhang

**Affiliations:** Department of Molecular Microbiology and Immunology, University of Texas at San Antonio, San Antonio, TX 78249, USA

**Keywords:** *Coxiella burnetii*, type IV secretion system, icmE protein, virulence, pyroptosis, inflammasome, caspase 1, cytokines

## Abstract

*Coxiella burnetii* is an obligate intracellular Gram-negative bacterium that causes Q fever, a life-threatening zoonotic disease. *C. burnetii* replicates within an acidified parasitophorous vacuole derived from the host lysosome. The ability of *C. burnetii* to replicate and achieve successful intracellular life in the cell cytosol is vastly dependent on the Dot/Icm type 4B secretion system (T4SSB). Although several T4SSB effector proteins have been shown to be important for *C. burnetii* virulence and intracellular replication, the role of the icmE protein in the host–*C. burnetii* interaction has not been investigated. In this study, we generated a *C. burnetii* Nine Mile Phase II (NMII) mutant library and identified 146 transposon mutants with a single transposon insertion. Transposon mutagenesis screening revealed that disruption of *icmE* gene resulted in the attenuation of *C. burnetii* NMII virulence in SCID mice. ELISA analysis indicated that the levels of pro-inflammatory cytokines, including interleukin-1β, IFN-γ, TNF-α, and IL-12p70, in serum from Tn::icmE mutant-infected SCID mice were significantly lower than those in serum from wild-type (WT) NMII-infected mice. Additionally, Tn::icmE mutant bacteria were unable to replicate in mouse bone marrow-derived macrophages (MBMDM) and human macrophage-like cells (THP-1). Immunoblotting results showed that the Tn::icmE mutant failed to activate inflammasome components such as IL-1β, caspase 1, and gasdermin-D in THP-1 macrophages. Collectively, these results suggest that the icmE protein may play a vital role in *C. burnetii* virulence, intracellular replication, and activation of inflammasome mediators during NMII infection.

## 1. Introduction

*Coxiella burnetii* is an obligate intracellular bacterium that causes the worldwide zoonotic disease, Q fever, in humans [1]. Acute Q fever manifests as a flu-like illness that is characterized by high fever, fatigue, body aches, and headaches; however, some acute infections can develop into more severe chronic diseases [2]. Chronic Q fever commonly presents as endocarditis [3], and more than 25% of untreated chronic Q fever infections in patients can be fatal [4]. Doxycycline treatment is more effective in alleviating acute infections if treatment occurs within three days of exposure, but chronic diseases are much more difficult to treat with antibiotics, usually requiring a combination of doxycycline and hydroxychloroquine to treat patients with chronic Q fever for at least 18 months [5,6]. *C. burnetii* is highly infectious and commonly spreads to humans via inhalation of contaminated aerosols generated from infected ruminants; therefore, Q fever is considered an occupational hazard among livestock workers and veterinarians [7,8]. Due to *C. burnetii*’s highly contagious nature and potential for fast dissemination, *C. burnetii* infection poses a threat to public health as well as having economic concerns [2]. This organism has also classified as a Tier 2 Select Agent due to its highly infectious, aerosol transmission in nature and resistance to adverse environmental conditions by the CDC [9].

Mononuclear phagocytes, such as alveolar macrophages, are the primary targets of *C. burnetii* [10]. Pulmonary alveolar macrophages phagocytose *C. burnetii*, which then transits canonically through the endolysosomal vesicular pathway [11]. *C. burnetii* become metabolically active once they reach an acidic environment and start to manipulate the vacuolar environment to support their replications [12]. This bacterially modified, spacious, and replicative membrane-bound compartment is termed the *Coxiella*-containing Vacuole (CCV) [13,14]. 

*C. burnetii* employs a Dot/Icm T4SSB system to survive, replicate, and thrive in acidified vacuoles [15]. T4SSB is essential for delivering over 100 bacterial effector proteins directly across the vacuole membrane into the cytosol of the host cell [14,16,17]. There is evidence that effector proteins translocated by T4SSB are very important for forming and maintaining vacuoles, being virulent, replicating bacteria, and controlling host cell death [18]. The deletion of T4SSB components in the genome leads to the total abolition of *C. burnetii* intracellular life, which highlights the importance of Dot/Icm T4SSB in *C. burnetii*. A few studies have already demonstrated the basic function of a few Dot/Icm T4SSB proteins, like icmL and icmD. These are inner membrane parts of T4SSB that are needed for infections and intracellular replication in host cells [19]. Furthermore, reports indicate that invasin and OmpA, two *Coxiella* T4SSB-independent novel virulence factors, are essential for invading epithelial cells, but not for invading macrophages [20]. Other effectors, like Cir, Cvp, and Ank, have been studied before and shown to be important. They are needed for the release of many other effectors [20,21,22]. Recent studies have also shown that the *C. burnetii* effector, IcaA, inhibits non-canonical inflammasome stimulation by inhibiting caspase 11 [20,23]. 

Although the characterization of other Dot/Icm Type IVB secretion system proteins has been documented [24], the role of the *C. burnetii* IcmE protein in host–pathogen interaction and in modulating the host immune response remains uninvestigated. In this study, a *C. burnetii* Nine Mile II (NMII) RSA 439 transposon genomic library was generated and a single transposon insertion in the icmE gene (Tn::icmE) mutant was identified by screening the *C. burnetii* NMII Himar1 transposon library. To characterize the role of icmE in *C. burnetii* during NMII infection, the NMII Tn::icmE mutant was used to examine whether (1) icmE is involved in *C. burnetii* NMII intracellular replication in mouse bone marrow-derived primary macrophages (MBMDM) and THP-1 macrophages in vitro; (2) icmE is responsible for NMII-induced disease in SCID Mice; and (3) icmE is required for NMII-infection-induced inflammatory responses in macrophages and SCID mice. Our results suggest that *C. burnetii* icmE protein may play an important role for *C. burnetii* intracellular replication, causing disease, and inducing inflammatory responses. 

## 2. Materials and Methods

### 2.1. Cultivation of C. burnetii 

This study utilized WT *C. burnetii* NMII (RSA 439, clone 4), along with its mutant strains, including Tn::dotA, Tn::icmE mutant and Tn::icmE complement. The *Coxiella* strains were cultivated in liquid acidified citrate cysteine medium (ACCM-D) using axenic growth methods, as previously reported [25,26]. Briefly, the stationary-phase bacteria were purified after 7 days cultivation and the pellets were resuspended in phosphate-buffered saline (PBS: 1.5 mM KH_2_PO_4_, 2.7 mM Na_2_HPO_4_⋅7H_2_O, 155 mM NaCl, pH 7.2). *C. burnetii* stocks were stored at −80 °C until use.

### 2.2. Mammalian Cell Culture

The THP-1 (TIB-202; ATCC, Manassas, VA, USA) and MBMDM were generated and cultivated in RPMI-1640 or DMEM medium (Gibco, Carlsbad, CA, USA) using the earlier established procedures [27,28]. THP-1 monocytes were differentiated into adherent macrophages using 200 nM phorbol 12-myristate 13-acetate (PMA; Sigma-Aldrich, Saint Louis, MO, USA). MBMDM were generated from adult female C57BL/6 mice (8–12 weeks-old, Jackson Laboratories, Bar Harbor, ME, USA). Macrophage progenitor cells were cultured in DMEM; containing 10% fetal bovine serum (FBS; Gibco), and recombinant murine macrophage colony-stimulating factor (rmM-CSF) at 20 ng/ml (Peprotech, Cranbury, NJ, USA) in 75-cm^2^ flasks. After 4 days, adherent cells were collected, replated in 24-well plates, and cultured overnight in fresh medium containing rmM-CSF. Both THP-1 and MBMDM were infected with WT NMII or transposon mutant strains included Tn::icmE, Tn::icmE complement, and Tn::dotA at a multiplicity of infection (MOI) of 100, as previously described [28,29,30]. 

### 2.3. Generation of C. burnetii NMII Mutant Library and Identification of Mutant Clones

Briefly, *C. burnetii*-specific transposon plasmid pITR-CAT-ColE1-P311 (a gift from Dr. Robert Heinzen and Dr. Paul Beare, National Institute of Allergy and Infectious Diseases, USA), which encodes a Himar1 transposase, was introduced into stationary-phase *C. burnetii* NMII using electroporation at 18 kV, 500 Ω, and 25 µF, following the protocols as described previously [31,32,33]. Following electroporation, the bacteria were enriched in 3 mL of ACCM-D medium for 24 h and 150 µL of electroporated culture was plated on ACCM-D agarose plates containing tetracycline. After seven days of incubation, single colonies were selected and grown in 3 mL of tetracycline containing ACCM-D in 24-well plates. The transposon insertion locus in the genome of each mutant was determined by two rounds of polymerase chain reaction (PCR). Primer 3 (TCGATTTTTGTGATGCTCGTC) was used to sequence the second PCR product. Sequencing data were analyzed and annotated using BLAST [20].

### 2.4. In Silico Analysis of Coxiella icmE Nucleotide and Protein Sequence

The icmE nucleotide and protein sequences were analyzed by bioinformatic annotation, according to the approaches as described previously [34,35]. Domain and motif profiles were analyzed using the SMART program (http://smart.embl-heidelberg.de/) (accessed on 20 May 2023). Multiple sequence alignment of the *Coxiella* icmE protein sequence with those of other bacterial species was performed using ClustalW 2 (http://www.ebi.ac.uk/Tools/msa/clustalw2/) (accessed on 20 May 2023). The 3D protein structure of *Coxiella* icmE (Accession Number, CBU-1627) was predicted using the online I-Tasser server (http://zhanglab.ccmb.med.umich.edu/I-TASSER) (accessed on 20 May 2023) and viewed using Pymol 3.0 analysis software. 

### 2.5. Complementation Assay 

The complementation of icmE was performed by using previous established techniques [20,33,36]. Briefly, the *Coxiella icmE* gene was inserted into the *SalI* cloning site of the *C. burnetii* complementation plasmid pJB-Kan:3xFLAG. Subsequently, a quantity of 10 μg of this construct was introduced into the stationary phase of *C. burnetii* Tn::icmE by electroporation at 18 kV, 500 Ω, and 25 µF. The bacteria were cultured in liquid ACCM-D medium without arginine and with kanamycin (350 μg/mL) for 4 days to select positive transformants. 

### 2.6. Intracellular Replication of Tn::icmE in THP-1 and Mouse BMDM by Immunofluorescence Microscopy 

THP-1 and MBMDM cells were infected with WT NMII or transposon mutant strains at MOI of 100. The cells were subjected to indirect immunofluorescence staining using the method described previously [20,33,36]. NMII-infected and uninfected cells serve as the positive and negative controls, respectively. The cells were stained with rabbit anti-*C. burnetii* polyclonal antibody and anti-LAMP-1 monoclonal antibody (Invitrogen, Carlsbad, CA, USA) for 60 minutes (min) at room temperature (RT). Furthermore, samples were incubated for 60 min at RT with respective secondary antibodies (1:3000 dilution) conjugated to R-phycoerythrin goat anti-rabbit IgG, Alexa Fluor 488 anti-CD107a (LAMP-1), and the host DNA stained with Hoechst 33342. Cells were imaged and captured using a Zeiss LSM-710 confocal fluorescence microscope and the images and fluorescence intensity profiles were analyzed using Zen software [20,29]. In addition, stained cells were visually analyzed for CCV formation in both THP-1 and MBMDM cells after being infected with the WT NMII, or transposon mutant strains that included Tn::icmE, Tn::icmE complement, and Tn::dotA, as previously published [11].

### 2.7. Intracellular Replication of Tn::icmE in THP-1 and Mouse BMDM by RT-qPCR

The THP-1 and MBMDM monolayers were infected with WT NMII or transposon mutant strains at MOI 100. NMII and uninfected cells serve as the positive and negative controls, respectively. Briefly, following a 3-day incubation period, cells were lysed with 200 µL lysis buffer (1 M Tris, 0.5 M EDTA, 7 mg/mL glucose, 28 mg/mL lysozyme) and 10 µL proteinase K. The lysate was collected, and genomic DNA was isolated from the samples using the Roche High Pure PCR Template Preparation Kit (Millipore Sigma, Burlington, MA, USA). Purified DNA was used as a template to quantify genomic equivalents (GE) by *com1* gene specific quantitative real-time PCR (RT-qPCR), as described previously [26,37]. 

### 2.8. Cytokine Analysis in Mouse BMDM Culture Supernatant

Cytokines produced by MBMDM in response to infection with WT NMII, or mutants at MOI of 100 were measured using a bead-based MAGPIX Luminex xMAP instrument and kit (Luminex^®^, Austin, TX, USA) according to the manufacturer’s instructions and previous reports [38,39]. The positive and negative controls are NMII and uninfected cells, respectively. Briefly, 25 µL of antibody magnetic beads directed against cytokines including IFN-γ, IL-1β, IFN-γ, TNF-α, TGF-β1, and IL-12p70 were loaded into each well in a black 96-well plate. The antibody beads were washed two times with 200 µL of wash solution. Then, 50 μL of the infected MBMDM culture supernatants and standards were added into the sample and standard wells respectively. The next day, biotinylated detection antibodies were added to the plate and incubated at RT with shaking for 60 min. Streptavidin-PE was then added and incubated at RT for 30 min in a rocker. The concentration of cytokines in the samples, standards, and blank controls were read in MAGPIX™ instrument. For data analysis, multiplex analyst software was used (Luminex 2.0). 

### 2.9. Immunoblotting 

Inflammasome protein expression was evaluated in THP-1 cells lysate by immunoblotting after being infected with WT *C. burnetii* NMII, or transposon mutants at MOI of 100, as described previously [29,30,40]. NMII and uninfected cells were used as the positive and negative controls, respectively. In summary, after 48 h, infected cells were lysed in 100 µL RIPA lysis buffer (50 mM Tris, 5 mM EDTA, and 1% sodium dodecyl sulfate). The total protein (10 μg/lane) in the supernatant was separated by 12% sodium dodecyl sulfate-polyacrylamide gel electrophoresis (SDS-PAGE) and transferred onto a 0.2 m pore polyvinylidene fluoride (PVDF) membrane (Thermo Fisher, Waltham, MA, USA) [30]. The membrane was blocked with 5% skim milk in Tris-buffered saline (150 mM NaCl, 100 mM Tris-HCl, pH 7.6) containing 0.1% Tween 20 (TBST) for 60 min at RT. The membrane was incubated overnight at 4 °C with rabbit polyclonal antibodies directed against caspase 1 (Cell Signaling, Danvers, MA, USA), IL1-β (Cell Signaling, Danvers, MA, USA), and gasdermin (Cell Signaling) in 5% non-fat skim milk or 5% BSA. The membrane was then incubated for 60 min at RT in TBST buffer containing anti-rabbit IgG secondary antibody conjugated to horseradish peroxidase (HRP). Protein expression was visualized using enhanced chemiluminescence substrates, such as urea and stable peroxide (Thermo Fisher, Waltham, MA, USA). The blot images were acquired using an iBright 1500 Imaging System (Invitrogen, Carlsbad, CA, USA) [29,40]. 

### 2.10. Virulence Determination in SCID Mice 

SCID mice (6-to-8 weeks-old female) were purchased from Jackson Laboratories and acclimatized in the University of Texas at San Antonio (UTSA) BSL-3 animal facility. All animal procedures were performed by the Animal Care and Use Committee (Animal Use Protocol, MU/CP-001, UTSA, San Antonio, TX, USA). Briefly, SCID mice were infected with 1 × 10^9^ *com1* gene copy GE of the WT NMII or mutants that included Tn::icmE, Tn::icmE complement, and Tn::dotA via intraperitoneal injection. The positive and negative controls are NMII and Tn::dotA-infected mice, respectively. After a 14-day infection period, the severity of virulence induced by WT NMII or transposon mutants in SCID mice was assessed by comparing the amount of body weight loss, splenomegaly, and bacterial burden in the spleen, as described previously [41,42,43,44]. The bacterial burden was assessed by TaqMan qPCR and expressed as *com1* gene copies in the whole spleen of each infected mouse [22].

### 2.11. Quantitation of Cytokines in SCID Mouse Serum 

The cytokines, including IL-1β, IFN-γ, TNF-α, TGF- β1 and IL-12p70, in serum of SCID mice infected with WT NMII, or transposon mutants were quantified using a bead-based MAGPIX Luminex xMAP instrument and kit (Luminex), according to the manufacturer’s instructions. Sera from NMII-infected and uninfected mice served as the positive and negative controls, respectively. Serum samples were collected from each SCID mouse infected with WT NMII or mutants and centrifuged to remove cellular debris. Cytokine’s level was measured in the serum as described above in Section 2.8. 

### 2.12. Data Analysis

All statistical analyses were performed using unpaired *t*-test in GraphPad Prism software (version 9.0) (GraphPad Software, San Diego, CA, USA). The statistical significance difference was determined as * *p* < 0.05, ** *p* < 0.01, and *** *p* < 0.001. 

## 3. Results

### 3.1. Generation of an Arrayed Mutant Library of C. burnetii NMII and Identification of a Himar1 Transposon Insertion in icmE

**Figure 1:**
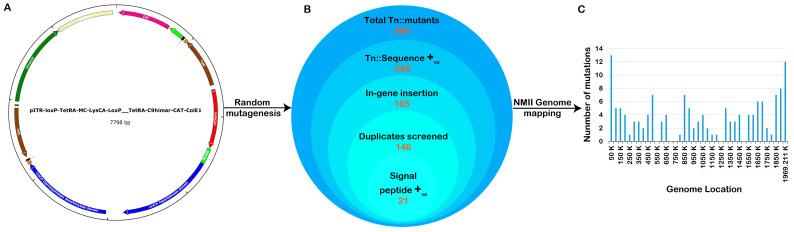
Construction of a *C. burnetii* NMII RSA439 transposon mutant library. (**A**) The pITR-CAT-ColE1-P311 transposable plasmid was employed to generate a *Coxiella* mutant library containing the tetracycline-resistant gene under the regulation of the *Coxiella* promoter p1169, flanked by Inverted Terminal Repeats (ITR). The blue curved arrow indicates the site of the nucleotide sequence encoding a lysine for autotropic-based selection. (**B**) Schematic presentation of the total number of transposon mutants obtained, trimmed, and annotated in the *Coxiella* NMII RSA439 genome. (**C**) The bar graph represents the location of each transposon insertion in the genome, and the bar height shows the number of mutants with a transposon insertion at each site.

Several previously published axenic protocols were used to perform genetic transformation, library construction, and clonal isolation of *C. burnetii* NMII transformants [19,31]. The pITR-CAT-ColE1-P311 plasmid, which contains a Himar1 transposase and tetracycline resistance gene, was introduced into the genome of the *C. burnetii* NMII strain RSA 439 in a random manner (Figure 1A). Following sequence annotation, a total of 364 transposon mutant colonies were identified. The transposon insertion site of each mutant onto the *C. burnetii* RSA 439 genome was determined using advanced techniques such as single-primer colony PCR and insertional sequencing mapping technology. Out of the 364 mutants, a significant number of 248 showed transposon insertions in the coding region of *C. burnetii* (Figure 1B). Following optimization, a total of 146 transformants were identified as single transposon insertion clones from 10 independent pools. Through bioinformatics annotation, it was discovered that 21 isolated clones contained transposon insertions in genes responsible for signal peptides, while 9 clones had transposon insertions in genes related to the Dot/Icm system. Figure 1C illustrates the distribution of mutants with transposon insertions across the *C. burnetii* genome. Using auxotrophic and antibiotic selection conditions, all clones were arranged and grown in liquid ACCM-D. Through annotation, we revealed a mutant with a transposon insertion in the gene responsible for translocating the T4SSB effectors, icmE. For this study, we utilized the Tn::icmE mutant to gain insights into the crucial function of icmE in *Coxiella* pathogenesis, intracellular replication, and the regulation of inflammatory responses. We employed both in vivo and in vitro models to investigate these aspects (Table 1). 

### 3.2. In Silico Analysis of the icmE Nucleotide and Protein Sequences

**Figure 2:**
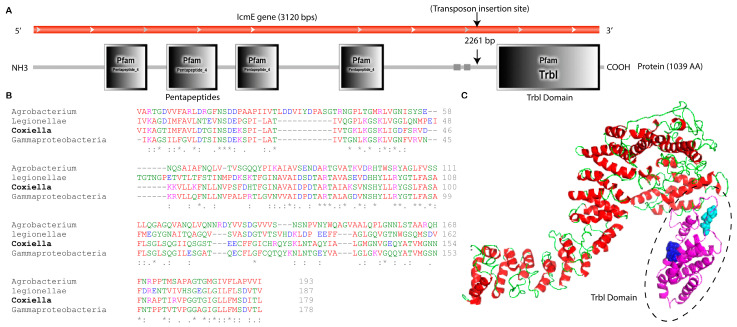
Bioinformatics analysis of *C. burnetii* icmE nucleotide and protein sequences. (**A**) Location of transposon insertions that disrupted icmE in the *C. burnetii* NMII RSA439 genome. The *C. burnetii* icmE gene is represented in the red frame, and the deduced protein and its domain profile are shown in the black solid frame. (**B**) Multiple sequence alignment of the TrbI domain of *C. burnetii* icmE with its orthologs. Strongly conserved sequences are highlighted by * symbols. (**C**) The predicted 3D structure of the *C. burnetii* icmE protein was viewed using PyMOL. The TrbI domain was highlighted in a dotted circle, and conserved motifs such as ‘NSD’ (purple) and ‘ART’ (sea blue) are shown in the ball model.

The icmE gene in *C. burnetii* is made up of 3120 base pairs (bp), which encode for a protein with 1039 amino acids, a molecular mass of 106.5 kDa, and an isoelectric point of 9.14. DNA sequencing and SMART domain profile analysis revealed the insertion of the transposon into the C-terminal domain of icmE, also known as the Type IV secretion protein domain (TrbI), situated between positions 851 and 1029 (Figure 2A,C). Researchers have reported that the Trbl domain, an inner membrane periplasmic protein domain, plays a crucial role in the bacterial T4SSB system, delivering nucleoprotein complexes and proteins essential for substrate secretion and conjugation [45]. Multiple sequence alignment analysis revealed that other bacteria, including *Legionella*, Gamma proteobacteria, and *Agrobacterium* species, were highly conserved with the amino acid composition of the TrbI domain of *C. burnetii* icmE (Figure 2B). There was a similarity between three groups of amino acids in the TrbI domain and the plant pathogen *Agrobacterium*. These groups included “NSD” and “ART” (Figure 2B). The Trbl domain in the *Coxiella* icmE protein may contribute to structural and functional properties that are critical for the conjugation and transportation of *C. burnetii* effector proteins across the host cytosol. As a result, transposon insertion in the TrbI domain of icmE might make this protein less functional in the pathogenesis of *C. burnetii*.

### 3.3. Tn::icmE Exhibits an Intracellular Replication Defect in Both MBMDM and THP-1-Derived Human Macrophages

**Figure 3:**
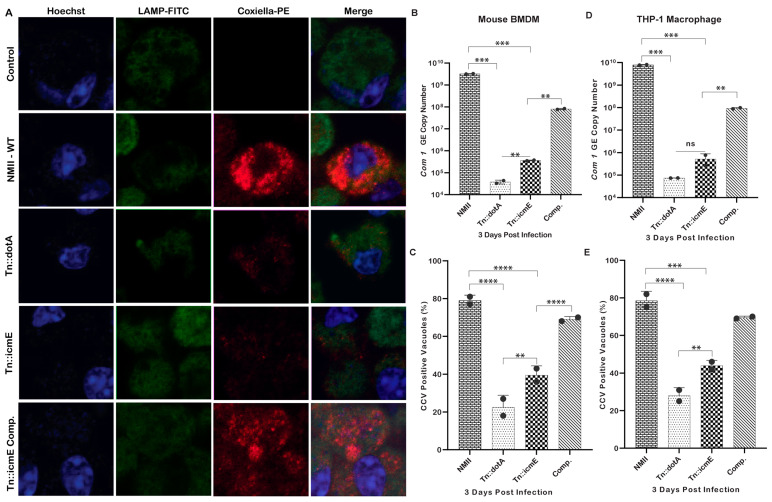
*C. burnetii* icmE is required for intracellular replication in MBMDM and THP-1 cells. The transposon icmE mutant was evaluated for its intracellular replication ability in MBMDM. MBMDM were infected with 100 MOI of WT NMII, Tn::dot, Tn::icmE or Tn::icmE complement in poly D-lysine-coated glass slide chambers. Following the infection for 3 days, all cells were fixed and stained with antibodies against *Coxiella* [46], LAMP1 (green) and Hoechst dye (blue) and examined by confocal fluorescence microscopy at magnification of 40×. (**A**) Confocal images of bacterial intracellular replication of WT NMII, Tn::dotA, Tn::icmE and Tn::icmE complement strains in MBMDM. *C. burnetii* bacterial numbers in MBMDM (**B**) and THP-1 macrophages (**D**) were determined by real-time qPCR and are expressed as *C. burnetii com1* gene copy numbers. In terms of *com1* gene copy number in genomic equivalent at 3 dpi. Percentage of vacuole formation in MBMDM (**C**) and THP-1 macrophages (**E**) at 3 dpi. Error bars indicate the mean ± standard deviation, and the results are expressed as the mean of three individual experiments, conducted with biological duplicates and three technical replicates. The *p*-value was calculated using an unpaired *t*-test. *p* < 0.01 **; *p* < 0.001 ***; and *p* < 0.0001 ****.

Previous studies have been shown that *Coxiella* mutants with transposon insertions in certain T4SSB genes, such as *icmD* or *icmL*, and complete deletions of *dotA* or *dotB* genes, are defective for bacterial intracellular replication [19,24]. These data demonstrate the essential role of T4SSB in the pathogenesis of *C. burnetii*. To determine whether *C. burnetii* icmE is necessary for bacterial intracellular replication, we compared the intracellular replication abilities among WT NMII, Tn::dot, Tn::icmE and Tn::icmE complement in MBMDM and THP-1 macrophages using a qualitative immunofluorescence microscopy and a qPCR assay. The results of an immunofluorescence microscopy assay showed that compared to WT NMII and complemented Tn::icmE strains, both Tn::icmE and Tn::dotA mutant strains exhibited growth defects in both MBMDM (Figure 3A) and THP-1 macrophages. Similarly, compared to WT NMII-infection-induced CCVs (75–85%), both Tn::icmE- (35–40%) and Tn::dotA- (25–30%) mutant infections induced very few CCVs in MBMDM (Figure 3C) and THP-1 macrophages (Figure 3E). Additionally, the CCVs formed within cells by the Tn::icmE mutant strain harbored fewer bacteria compared to the CCVs formed by the WT NMII strain. Furthermore, compared to WT NMII or Tn::icmE-complement-infected MBMDM and THP-1 macrophages, the *com1* gene copy number was significantly lower in Tn::icmE or Tn::dotA-infected MBMDM (Figure 3B) and THP-1 macrophages (Figure 3D). These results indicate that icmE is required for NMII bacterial intracellular replication. Collectively, these results suggest that *C. burnetii* icmE plays an essential role for bacterial intracellular replication and CCV formation in both MBMDM and THP-1 macrophages (Table 2).

### 3.4. Cytokine Response in MBMDM Supernatant

**Figure 4:**
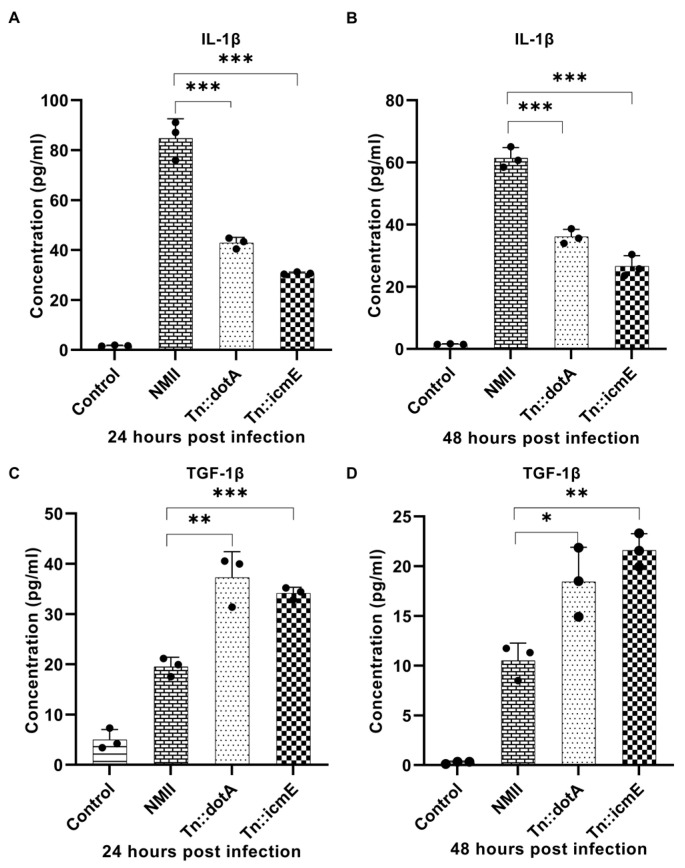
Compare WT NMII-, Tn::dotA- and Tn::icmE-infection-induced cytokine responses in MBMDM. MAGPIX Luminex analysis of IL-1β and TGF-β1 cytokines in culture supernatants from MBMDM infected with WT NMII, Tn::dotA, or Tn::icmE at MOI of 100 at 24 and 48 hpi. Compared to WT NMII-infected cells, Tn::icmE or Tn::dotA-infected cells had lower levels of IL-1β secretion (**A**,**B**) at 24 and 48 hpi, and increased the level of TGF-β1 secretion (**C**,**D**) at 24 and 48 hpi. Error bars indicate the mean ± standard deviation, and the results are expressed as the mean of three individual experiments, conducted with biological duplicates and three technical replicates. The *p*-value was calculated using an unpaired *t*-test. *p* < 0.05 *; *p* < 0.01 **; and *p* < 0.001 ***.

Murine primary macrophages have been used to investigate host innate immune responses to *C. burnetii* infection [18]. To understand the early stage of macrophage response to *C. burnetii* infection, we examined the release of cytokines in the supernatants from MBMDM infected with WT NMII, Tn::icmE or Tn::dotA strain at 24 and 48 h post infection. The supernatants were collected from BMDM infected with 100 MOI of WT NMII, Tn::icmE or Tn::dotA strain at 24- and 48-h post-infection (hpi). Compared to the WT NMII-infected MBMDM, the Tn::icmE and Tn::dotA infections resulted in increased secretion of TGF-β1 (Figure 4C,D) and reduced the level of IL-1β (Figure 4A,B) in MBMDM. These results indicated that the Tn::icmE mutant was unable to reduce the release of pro-inflammatory cytokine but increase the level of anti-inflammatory cytokine in MBMDM. The decreased secretion of cytokines might be due to the lack of bacterial intracellular replication in the Tn::icmE or Tn::dotA-infected MBMDM, suggesting that a functional T4SSB is required for triggering the host innate immune response against *C. burnetii* infection. 

### 3.5. Role of C. burnetii icmE in Basic Inflammasome Component Activation in THP-1 Macrophages

**Figure 5:**
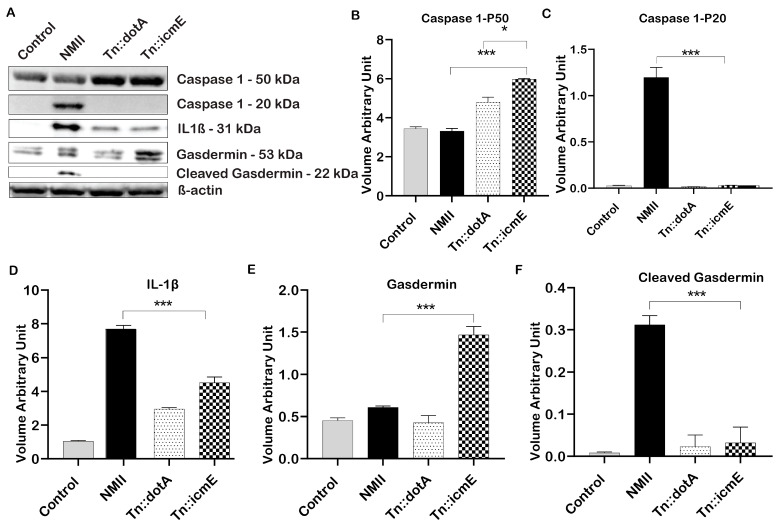
***C****. burnetii* icmE protein is required for activation of caspase 1-dependent inflammasome in THP-1 macrophages. THP-1-derived macrophages were infected with uninfected or infected with WT NMII, Tn:icmE or Tn::dotA at an MOI of 100. (**A**) Immunoblot of the inflammasome pathway-related proteins, caspase 1, cleaved caspase 1, IL-1β, Gasdermin and cleaved Gasdermin in the supernatants from uninfected, WT NMII-, Tn::icmE- or Tn::dotA-infected THP-1 macrophages at 48 hpi. Relative intensity unites of caspase 1 (**B**), cleaved caspase 1 (**C**), IL-1β (**D**), Gasdermin (**E**) and cleaved Gasdermin (**F**). The *p*-value was calculated using an unpaired *t*-test. *p* < 0.05 *; and *p* < 0.005 ***. The symbols *p* < 0.05, *, and *p* < 0.001, *** are used to denote the presence of significant differences across samples, as determined by the unpaired *t*-test. The error bars represent the standard deviations of the means. The experiment was performed in triplicate.

In order to explore the role of icmE in activation of inflammasome during *C. burnetii* infection in human macrophages, the expression of proteins that involved in activation of inflammasome in WT NMII-, Tn::icmE- or Tn::dotA-infected THP-1 macrophages was analyzed by immunoblotting at 48 hpi. As shown in Figure 5A, cleaved caspase 1 (20 kDa) and GSDMD (22 kDa) were detected in WT NMII-infected THP-1 macrophages but did not detect in Tn::icmE- and Tn::dotA-infected THP-1 macrophages. In addition, a larger amount of IL-1β was detected in WT NMII-infected THP-1 macrophages than in Tn::icmE- and Tn::dotA-infected THP-1 macrophages. The intensity units of the cleaved caspase 1 (Figure 5C), cleaved GSDMD (Figure 5D) and IL-1β (Figure 5F) protein bands in WT NMII-infected THP-1 macrophages were significantly higher than those in uninfected, Tn::icmE- and Tn::dotA-infected THP-1 macrophages. These results indicated that both Tn::icmE and Tn::dotA mutant strains were unable to activate caspase 1-dependent inflammasome in human macrophages, suggesting a functional T4SSB system and bacterial replication maybe required for activation of caspase 1-dependent inflammasome in human macrophages. The activation of inflammasomes plays a critical role in facilitating the efficient innate inflammatory responses against intracellular bacterial pathogens, which result in pyroptosis and the limitation of pathogens [19]. *C. burnetii* seems similar to other intracellular bacteria, can exert an influence on innate immune responses by T4SSB, and consequently can affect the components of inflammasomes [18,23,47]. 

### 3.6. Disruption of the icmE Gene Attenuates C. burnetii NMII Virulence in SCID Mice

**Figure 6:**
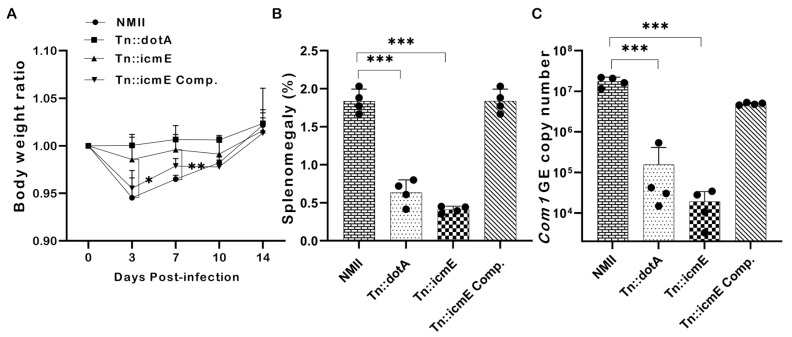
Evaluate the severity of Tn::icmE mutant infection-induced disease in SCID mice. The SCID mice were infected with 1 × 10^9^ GE of WT NMII, Tn::dotA, Tn::icmE or Tn::icmE complement. (**A**) Relative body weights (current body weight/day 0 body weight) were measured throughout the challenge period. (**B**) Splenomegaly (% of spleen weight/body weight). (**C**) bacterial burden in the spleen was determined by real-time qPCR and is expressed as *C. burnetii com1* gene copy numbers. Each experimental group consists of four mice, and the error bars indicate the standard deviations from the means. The symbol *p* < 0.05, *; *p* < 0.01, **; *p* < 0.001, *** was used to denote the presence of significant differences across samples, as determined by the unpaired *t*-test.

To examine if disruption of the icmE gene in NMII would affect its ability to cause disease, SCID mice were intraperitoneally infected with 1 × 10^9^ GE of WT NMII, Tn::dotA, Tn::icmE or Tn::icmE complement. Splenomegaly and bacterial burden in the spleen were examined at 14 dpi. The severity of Tn::icmE mutant infection-induced disease in SCID was evaluated by comparing body weight loss, splenomegaly, and bacterial burden in the spleens with WT NMII-, Tn::dotA- and Tn::icmE complement *Coxiella*-infected mice. As shown in Figure 6A, both Tn::icmE- and Tn::dotA-infected mice did not show any significant body weight loss during *C. burnetii* infection; however, WT NMII- and Tn::icmE complement-infected mice exhibited significant body weight loss at 3 and 7 dpi. In addition, splenomegaly (Figure 6B) and bacterial burden in the spleens (Figure 6C) in Tn::icmE- and Tn::dotA-infected mice were significantly lower than in WT NMII- and Tn::icmE complement-infected mice. These results indicated that both Tn::dotA and Tn::icmE mutant strains lost their abilities to cause disease and replicate in SCID mice, suggesting that a functional T4SSB system is required for *C. burnetii* NMII strain to replicate and cause disease in SCID mice. 

### 3.7. Cytokine Secretion in Serum Samples from WT NMII-, Tn::dotA- and Tn::icmE Complement Strain-Infected SCID Mice 

**Figure 7:**
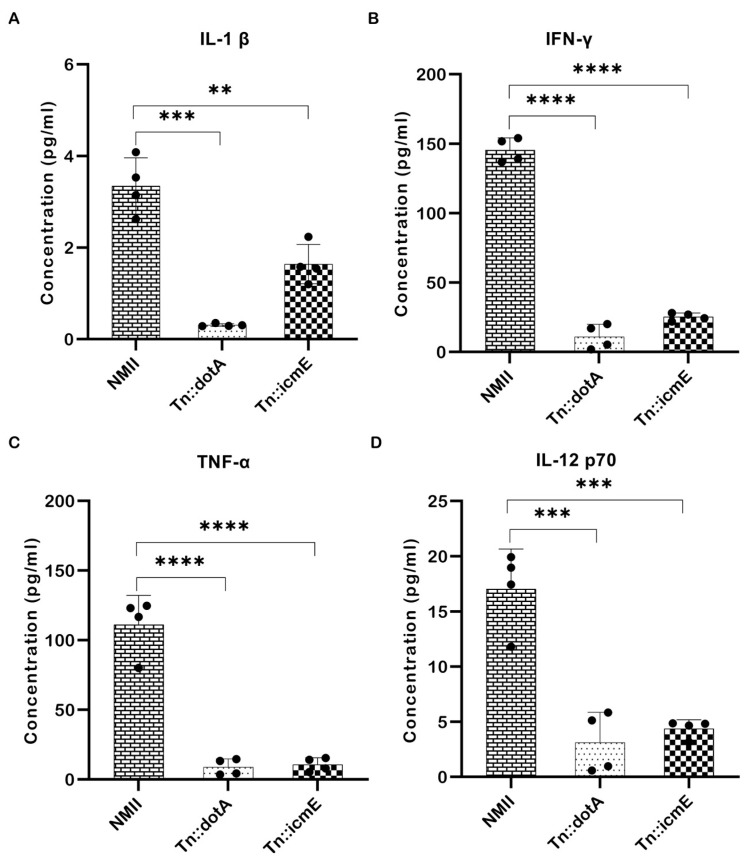
The Tn::icmE mutant induced lower levels of pro-inflammatory cytokine responses in SCID mice. The SCID mice were infected intraperitoneally with 10^9^ GE of WT NMII, Tn::dotA or Tn::icmE. Serum cytokine concentrations of IL-1β (**A**), IFN-γ (**B**), TNF-α (**C**), and IL-12p70 (**D**) were measured using the MAGPIX Luminex xMAP instrument at 14 dpi. Each experimental group consists of four mice, and the error bars indicate the standard deviations from the means. The symbols *p* < 0.01,**, *p* < 0.001,***, and *p* < 0.0001,**** are used to denote the presence of significant differences across samples, as determined by the unpaired *t*-test.

Pro-inflammatory cytokines play an important role in host defense against intracellular microbial infections. They are produced during the early stages of infection and have a role in multiple aspects of the host’s inflammatory and immunological response [19]. To determine the importance of icmE in stimulating systemic inflammatory cytokines in host in vivo, the concentrations of pro-inflammatory cytokines is serum samples from WT NMII-, Tn::dotA- and Tn::icmE complement strain-infected SCID mice were measured at 14 dpi. As shown in Figure 7, the concentrations of IL-1β (Figure 7A), IFN-γ (Figure 7B), TNF-α (Figure 7C), and IL-12p70 (Figure 7D) in Tn::icmE- and Tn::dotA-infected mice were significantly lower than in WT NMII-infected mice. These results indicate that both Tn::dotA and Tn::icmE mutant strains induced lower levels of pro-inflammatory cytokine responses than WT NMII strains in SCID mice, suggesting that a functional T4SSB system is required for *C. burnetii* NMII strain infection-induced inflammatory responses.

## 4. Discussion

According to the existing reference genomes and previous research on *Coxiella*, it has been shown that only a small number of Dot/Icm T4SSB system proteins are essential for *C. burnetii* intracellular survival and replication, and its ability to cause disease and to modulate host cell signaling pathways [19,24]. The present study provided first evidence to demonstrate that *C. burnetii* icmE is required for NMII bacterial intracellular survival and replication, and its ability to cause disease and induce pro-inflammatory cytokine responses in immunodeficient SCID. A *C. burnetii* NMII RSA 439 transposon genome library was generated, and a total of 146 mutants with a single insertion were identified. In addition, the utilization of clonal isolation and sequencing techniques has unveiled the presence of a transformant clone in *C. burnetii* that has a transposon insertion inside the Dot/Icm T4SSB protein, known as icmE. The analysis of *Coxiella* icmE nucleotides and protein sequences using bioinformatic approaches provided evidence that *C. burnetii* icmE contains a Trbl domain located at the C-terminus. Several studies have shown confirmation of the presence of the Trbl domain in several bacterial pathogens and demonstrated the importance of Trbl domain in pili production, conjugation, and the translocation of T4SSB effectors across membranes into the cytoplasm of host cells [45,48,49]. The sequence homology among the Trbl domain in *Coxiella* icmE and TrbI of *Legionella* icmE gene, the plasmid RK2, and VirB10 related proteins has been reported [45]. This finding suggests that the TrbI domain could be essential for the formation of stable structural and function properties of the *Coxiella* icmE protein, which may be responsible for its pathogenesis. 

*C. burnetii*, a bacterium characterized for its high level of infectivity, promotes replication in macrophages through the inhibition of several host cell immune responses [24]. Many studies have demonstrated the significance of T4SSB, and its released effectors play a pivotal role in facilitating the proliferation of *C. burnetii* in macrophages and permissive cells, hence promoting the survival of the bacteria [50]. In this study, we isolated the icmE clone, recovered and replicated in ACCM-D axenic medium. However, the Tn::icmE mutant strain showed significant impairments in intracellular growth in BMDM, THP-1, and SCID mice as compared to the WT NMII strain. The significance of the Dot/Icm secretion system in the pathogenesis of *C. burnetii* has been elucidated by the investigation of the phenotypic characteristics of mutants expressing Dot/Icm genes. The results presented in this study align with previous research conducted by Beare et al. [39] revealed that the deletion of dotA and dotB, along with transposon mutagenesis of dotA, dotB, icm V, D, G, J, N, C, P, K, X, and L1, effectively limit the establishment of the replicative niche and bacterial replication in host cells [19,24,51]. This result highlights the significant role of the *C. burnetii* icmE protein in its intracellular survival and replication. 

*C. burnetii* infection elicits the release of pro-inflammatory cytokines in both in vivo and in vitro conditions [18,52,53,54]. Significantly, T4SSB regulates the immunological response of the host, affects the interaction with macrophages, and results in the release of cytokines in the host cells [47,55,56]. Considering the absence of splenomegaly and the low bacterial burden in the spleens observed in SCID mice following *C. burnetii* icmE mutant infection, it is possible to suggest a hypothesis that the inability of the *C. burnetii* icmE mutant to replicate could be attributed to its inability to release effector proteins responsible for regulating host cell inflammatory cytokine signaling. To test this hypothesis, the concentrations of pro-inflammatory cytokines in serum samples from WT NMII-, Tn::dotA- and Tn::icmE complement strain-infected SCID mice were measured at 14 dpi. Interestingly, the concentrations of IL-1β, IFN-γ, TNF-α, and IL-12p70 in Tn::icmE- and Tn::dotA-infected mice were significantly lower than in WT NMII-infected mice. These results indicate that both Tn::dotA and Tn::icmE mutant strains induced lower levels of pro-inflammatory cytokine responses than WT NMII strains in SCID mice, suggesting that a functional T4SSB system is required for *C. burnetii* NMII strain infection-induced inflammatory responses.

Furthermore, we observed negligible IL-1β secretion in the culture supernatant of primary MBMDM after infection with Tn::icmE. Previous reports have demonstrated that pro-inflammatory cytokines such as IL-1β and TNF-α are overproduced in *C. burnetii*-infected patient monocytes and could be associated with a specific inflammatory syndrome of Q fever endocarditis [57,58,59,60]. This shows that virulent and avirulent *C. burnetii* regulate the pro-inflammatory cytokine signaling cascade in a T4SSB-dependent manner to escape the macrophage immune response [57,58,59]. Similar to our findings, Hu et al. [52] also demonstrated that infection with a virulent strain of *C. burnetii* in BALB/c mice elevated the serum levels of cytokines, including IL-1β, TNF-α, IFN-γ, and IL-12p70, suggesting an association between the expression of these pro-inflammatory cytokines and the virulence of *C. burnetii*. These observations led to the interpretation that the Tn::icmE mutant maybe exhibit a replication defect within host cells, resulting in the inability to induce a robust inflammatory response during the 14-day infection period. Consequently, deficiency of the *C. burnetii* icmE protein, leading to the loss of T4SSB effector proteins in *C. burnetii*, may prevent the induction of the host inflammasome signaling pathways required to produce pro-inflammatory cytokines, thereby evading host defense. 

In human alveolar macrophages, the activation of IL-1β specific to NMII involves the cleavage of caspase 5 and subsequent caspase 4, accompanied by increased expression of NLRP3. Notably, despite these molecular events, no cell lysis or death was observed [23,30,47,61,62]. Similarly, in murine B1a cells, WT NMII, but not Tn::dotA, induces caspase 1-mediated pyroptosis, regulated by the host Toll-like Receptor 2 (TLR2) and NLRP3 [30]. The results of this study showed that Tn::icmE and Tn::dotA mutant infections in THP1 macrophages did not affect the levels of inflammasome components, including caspase 1, IL-1β, and gasdermin D. This suggests that these mutants are unable to activate the inflammasome pathway. On the other hand, WT NMII exhibited cleavage of caspase 1 and gasdermin D, resulting in elevated levels of intracellular IL-1β. Previous studies have demonstrated that the IcaA protein reduces pore formation and inhibits inflammasomes and caspase 1 activation in *C. burnetii* [23]. Inhibition and activation of the inflammasome pathway have also been reported in other intracellular microbes, such as *Legionella* and *Francisella* [46,63].

The suitability of the SCID mouse model for evaluating the relative virulence nature of *C. burnetii* strains displaying phase I and II lipopolysaccharides by single and competitive infection experiments has been shown in previous report [43]. According to previous studies, SCID mice exhibit increased vulnerability to fatal infection caused by WT NMII strains of *C. burnetii* when exposed to intraperitoneal challenge [44,64]. Our study aimed to assess two fundamental virulence characteristics, namely splenomegaly and the capacity for splenic replication of Tn::icmE mutant. In comparison to WT NMII, both Tn::icmE and Tn::dotA mutants demonstrated a decrease in bacterial burden in the spleen and splenomegaly. This data demonstrates that the Tn::icmE mutant attenuated virulence and a growth defect in vivo, like the Tn::dotA mutant, whereas WT NMII exhibited high virulence in SCID mice. This finding aligns with a study conducted by Van Schaik et al. [1], wherein they observed that SCID mice infected with T4SSB dotA mutants displayed decreased splenomegaly and bacterial load in comparison to WT NMII mice. The observations presented underscore the crucial significance of icmE in the pathogenicity and replication of *C. burnetii* in host cells.

In summary, this study demonstrated previously unknown functions of the icmE protein using a transposon mutant in relevant macrophage cells and a mouse model. Our findings suggested that icmE *Coxiella* mutant did not induce splenomegaly in immunodeficient mice and in macrophages that lack intracellular replication, suggesting that icmE is necessary for *Coxiella* survival in host. Thus, the findings of this study establish a theoretical foundation for the development of new drugs and vaccines to combat *C. burnetii* and offer innovative therapeutic approaches for Q fever.

## Figures and Tables

**Table 1:** An overview of the NMII *C. burnetii* RSA 439 clone 4 mutants from random mutant library that were discovered in our study.

RSA439 NMII Random Library	Number of Mutant Clones
Total mutants obtained	364
Mutants with transposon insertion	248
Mutants with single transposon insertion	146
Transposon insertion in genes with signal peptide	21
Transposon insertion in Dot/Icm system genes	9

**Table 2:** In MBMDM and THP-1 cells, the percentage of CCV production following infection with WT NMII or mutants.

Treatments	CCV Percentage (%) in Mouse BMDM	CCV Percentage (%) in THP-1
WT NMII	79	78.5
Tn::dotA	22.5	28
Tn::icmE	31.5	44
Tn::icmE complement	69	69.5

## Data Availability

All data are comprised within this manuscript.
